# Non-Coding RNAs in Pediatric Solid Tumors

**DOI:** 10.3389/fgene.2019.00798

**Published:** 2019-09-20

**Authors:** Christopher M. Smith, Daniel Catchpoole, Gyorgy Hutvagner

**Affiliations:** ^1^School of Biomedical Engineering, University of Technology Sydney, Sydney, Australia; ^2^School of Software, University of Technology Sydney, Sydney, Australia; ^3^The Tumour Bank–CCRU, Kids Research, The Children’s Hospital at Westmead, Sydney, Australia

**Keywords:** pediatric tumors, miRNA, long noncoding RNA, cancer biology, gene expression

## Abstract

Pediatric solid tumors are a diverse group of extracranial solid tumors representing approximately 40% of childhood cancers. Pediatric solid tumors are believed to arise as a result of disruptions in the developmental process of precursor cells which lead them to accumulate cancerous phenotypes. In contrast to many adult tumors, pediatric tumors typically feature a low number of genetic mutations in protein-coding genes which could explain the emergence of these phenotypes. It is likely that oncogenesis occurs after a failure at many different levels of regulation. Non-coding RNAs (ncRNAs) comprise a group of functional RNA molecules that lack protein coding potential but are essential in the regulation and maintenance of many epigenetic and post-translational mechanisms. Indeed, research has accumulated a large body of evidence implicating many ncRNAs in the regulation of well-established oncogenic networks. In this review we cover a range of extracranial solid tumors which represent some of the rarer and enigmatic childhood cancers known. We focus on two major classes of ncRNAs, microRNAs and long non-coding RNAs, which are likely to play a key role in the development of these cancers and emphasize their functional contributions and molecular interactions during tumor formation.

Pediatric cancers are often categorized as hematologic, intracranial, or extracranial (Chen et al., 2015). Hematologic cancers include those derived from the blood or blood forming tissues, including bone marrow and the lymph nodes. Intracranial cancers are tumors that develop inside the brain, whereas extracranial solid tumors, often referred to as pediatric solid tumors, arise outside the brain. Collectively, pediatric solid tumors represent approximately 40% of all pediatric cancers and commonly form in the developing sympathetic nervous system (neuroblastoma), retina (retinoblastoma), kidneys (Wilms tumor), liver (hepatoblastoma), bones (osteosarcoma, Ewing sarcoma), or muscles (rhabdomyosarcoma) (Kline and Sevier, 2003; Allen-Rhoades et al., 2018). Solid tumors can originate from cells of any of the three germ layers, the ectoderm, mesoderm, or endoderm, and likely arise due to disruptions in the developmental processes of these precursor cells, leading them to develop cancerous phenotypes (Chen et al., 2015). This contrasts with most adult cancers, which tend to be of epithelial origin and are believed to develop over time due to exposure to toxins and environmental stress. As a result, adult cancers often display a high occurrence of genetic mutations, whereas pediatric solid tumors tend to feature a relatively low number of genetic mutations. This has led to investigations into alternative forms of gene regulation that may contribute to the emergence and development of cancerous cells in pediatric cancers.

Non-coding RNAs (ncRNAs) form a group of functional RNAs lacking protein-coding potential, which play a crucial role in the regulation of gene expression at every level, from epigenetic regulation via methylation and chromatin packaging to post-transcriptional regulation (Cech and Steitz, 2014; Zhao et al., 2016). The most widely studied ncRNAs are the microRNAs (miRNAs), small 20- to 25-nucleotide-long RNAs that play an important role in regulating translation and messenger RNA stability via complementary base pairing (Huang et al., 2011). Other classes of small ncRNAs include small interfering RNAs (siRNAs), Piwi-interacting RNAs (piRNAs), small transfer RNAs (tRFs), small nucleolar RNAs (snoRNAs), small nuclear RNAs (snRNAs), and small cytoplasmic RNAs (scRNAs). Additionally, long non-coding RNAs (lncRNAs) are a loosely defined group of RNAs normally larger than 200 nucleotides long that lack protein-coding potential and do not fall into any of the other categories but nonetheless play key roles in the regulation of gene expression (Mercer et al., 2009). Increasing data suggest that ncRNAs play a role in regulating all biological processes, and it is no surprise that studies have observed widespread dysregulation of ncRNAs in nearly all forms of cancer (Prensner and Chinnaiyan, 2011; Leichter et al., 2017). Interestingly, dysregulated RNA patterns are often specific to the type of cancer or even subtype and can provide insight into the mechanisms underlying phenotypic differences between tumors or cells within a tumor, such as their aggressiveness or resistance to certain types of treatments (Blenkiron et al., 2007; Li et al., 2014). Additionally, genome-wide association studies have suggested that over 80% of single nucleotide polymorphisms found associated with cancer are outside of coding regions (Carninci et al., 2005; Cheetham et al., 2013). In this review, we will discuss how two major classes of ncRNAs, miRNAs and lncRNAs, may contribute to pediatric solid tumors by participating in the regulation of established oncogenic networks known to drive these cancers.

## MicroRNAs and Gene Regulation

Not long after the first human miRNA, *let-7*, was discovered in 2002 by the Ruvkun lab, miRNAs began to emerge as key participants in tumorigenesis (Pasquinelli et al., 2000). In 2002, two miRNAs were identified as potential tumor suppressors due to their frequent downregulation or deletion in chronic lymphocytic leukemia (Calin et al., 2002). Calin et al. (2004) later showed that many miRNA genes are located close to fragile sites or common breakpoints that frequently occur in cancers, suggesting that their loss of function was a key event in oncogenesis. Since then, oncomiRs—cancer-associated miRNAs—have become a major research focus (Esquela-Kerscher and Slack, 2006). A better understanding of the mechanisms behind miRNA regulation in cancer is invaluable to researchers and clinicians alike, not only to aid in the identification of new drug targets but also for the development of promising RNA-based therapies and their potential use as early detection biomarkers.

### miRNAs: Biogenesis and Functions

The life cycle of a miRNA typically begins with its transcription into a primary miRNA (pri-miRNA) by RNA polymerase II (Ha and Kim, 2014). pri-miRNAs share several similarities with messenger RNAs (mRNAs) in that they are 5’ capped, are 3’ polyadenylated, and can be several hundreds or thousands of nucleotides long. In many cases, the pri-miRNA encodes for one miRNA species; however, in humans, a substantial number are polycistronic and encode several different miRNAs together. pri-miRNAs must be processed in the nucleus by the RNAse III enzyme Drosha, which releases shorter ∼65-nucleotide-long precursor RNAs (pre-miRNAs) with a secondary hairpin structure. This hairpin is recognized by the Exportin-5/Ran-GTP transporter, which transports the pre-miRNA from the nucleus to the cytoplasm. In the cytoplasm, the pre-miRNA is further processed by Dicer, another RNAse III enzyme, which cleaves the loop and releases a double-stranded miRNA duplex containing the 5 prime (5p) and 3 prime (3p) sequences. The duplex is then recognized by one of the four human Argonaute proteins, which loads one of the strands and discards the other.

miRNAs carry out their functions by binding to Argonaute and associating with various other proteins to form the RNA induced silencing complex (RISC). As part of this complex, miRNAs serve as guides by binding via complementary base pairing to target sites that are normally found in the 3´-untranslated region (3´UTR) of mRNAs. RISCs can regulate gene expression by direct cleavage of transcripts, transcript destabilization, or blocking translation. In a broader sense, miRNAs play a role in globally “fine-tuning” gene expression and are particularly important in inducing and maintaining differentiated cell states. In cancer, this finely tuned expression is often impaired, enabling gene networks that are normally switched on or off to reverse and begin influencing cellular behavior in a deleterious manner.

### miRNAs: Drivers or Passengers in Cancer?

Microarrays and next-generation sequencing technologies enabled global measurements of miRNA expression changes and have revealed miRNA dysregulation to be a hallmark in nearly all cancers. miRNA expression profiles often correlate with cancer subtypes and have been effective at classifying cancer samples for risk stratification (De Preter et al., 2011). However, understanding the contribution of specific miRNAs can prove difficult. miRNAs are predicted to regulate hundreds to thousands of genes; however, their influence may be minor, and often, they must act in concert with other miRNAs. Current miRNA target prediction algorithms are imperfect and do not capture the true range of regulatory targets; therefore, biological validation is still needed (Riffo-Campos et al., 2016). Additionally, opposing behavior is seen with many miRNAs, where the same miRNA may be considered an oncogene in one cancer and a tumor suppressor in another. Because of their integration within complex gene networks, it is often not obvious whether a dysregulated miRNA actively participates in the maintenance of a cancerous state or whether it is simply a bystander. Therefore, it is important to examine how miRNAs participate in oncogenic networks on a functional level in order to properly understand their role.

**Figure 1:**
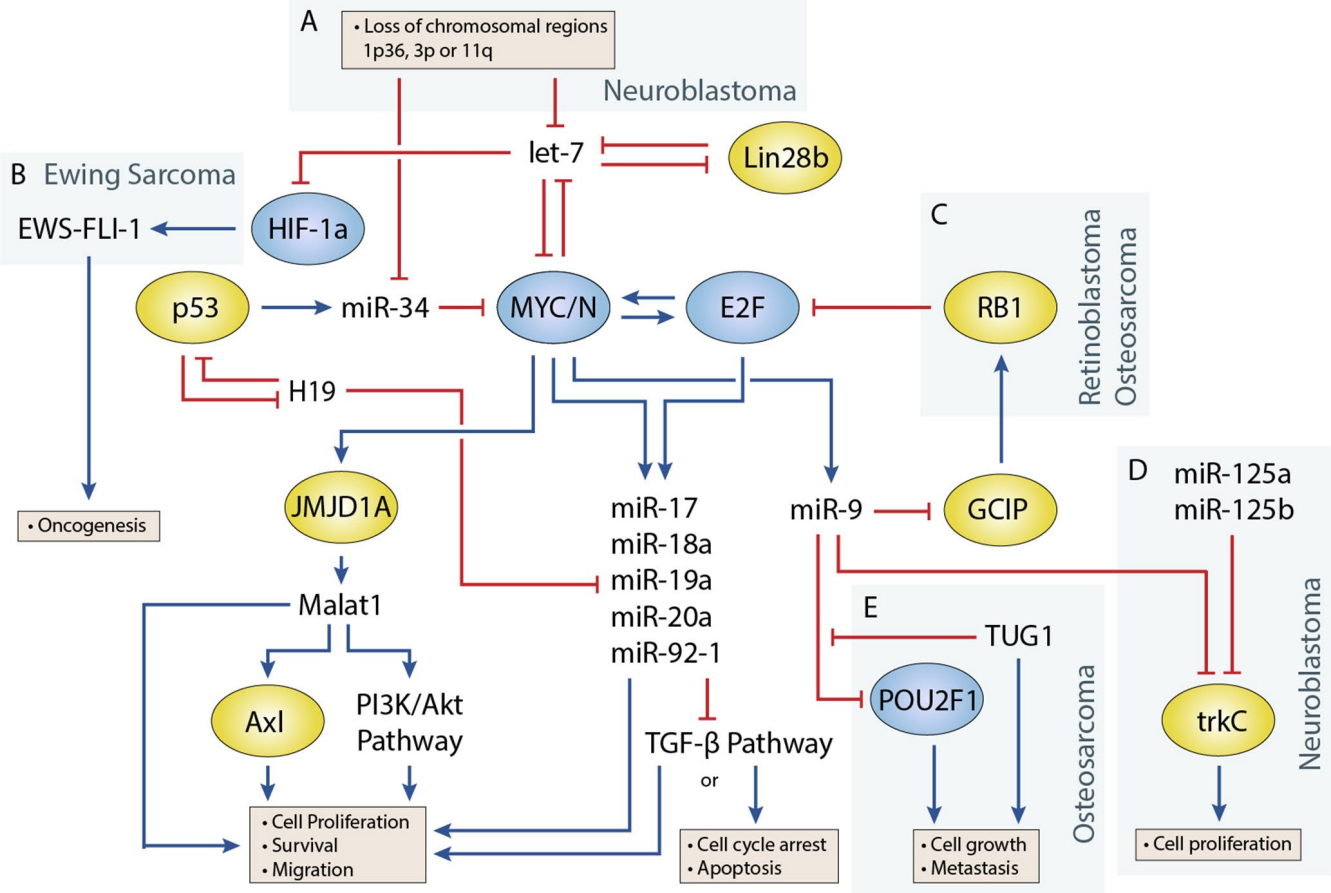
Regulatory circuitry involving non-coding RNAs in various pediatric solid tumors. Shows elements of a key regulatory circuit involving MYC and E2F family transcription factors and many ncRNAs, often dysregulated in pediatric solid tumors. In many cases, recurring dysregulation of specific elements, including miRNAs and lncRNAs, is observed and may represent vulnerabilities in the normal development of specific cell lineages. **(A)** Loss of chromosomal regions where let-7 and miR-34 miRNAs are localized is frequently observed in neuroblastoma and may represent a key event in the development of many of these cancers. **(B)** let-7 dysregulation may facilitate overexpression of the oncogenic fusion transcript EWS-FLI-1 in Ewing sarcoma. **(C)** The RB1 tumor suppressor regulates E2F, and loss of function via mutations can lead to the development of retinoblastoma. In osteosarcoma, miR-9 may be able to act as an oncogenic driver as it is often overexpressed and can downregulate RB1. **(D)** In neuroblastoma, miR-9 can display tumor-suppressive properties by cooperating with miR-125a and miR-125b to regulate a specific isoform of trkC and suppress cell proliferation **(E)** The lncRNA TUG1 is suggested to act as a ceRNA against miR-9, which has been shown to display tumor-suppressive properties in some osteosarcoma cell lines.

Transcription factors that play an important role in regulating cell proliferation, migration, and apoptosis are commonly perturbed in pediatric solid tumors. One of the best examples of this is in neuroblastoma, where *MYCN* amplification is present in approximately 25% of neuroblastoma patients and disproportionally represents high-risk cases (Huang and Weiss, 2013). MYCN upregulation is also observed at a higher frequency in several other pediatric solid tumors including Wilms tumor, rhabdomyosarcoma (Williamson et al., 2005), and retinoblastoma, although generally not to the extent seen in neuroblastoma. Germline inactivation of the Wilms Tumor 1 (WT1) transcription factor has been linked to a genetic predisposition towards Wilms tumor. Several transcription factors, including Twist, Snails, and Zebs, involved in the epithelial-to-mesenchymal transition have also been implicated in the development of osteosarcoma (Yang et al., 2013). miRNAs are often closely tied to transcription factors, either as regulators or as transcriptional targets (**Figure 1**) (Sin-Chan et al., 2019). One of the earliest studies linking miRNAs to an oncogenic transcription factor was by O’Donnell et al. in 2005 (O’Donnell et al., 2005). In this study, they demonstrated that c-Myc could induce expression of the miR-17∼92 cluster and that several of these miRNAs could in turn regulate E2F1 transcription to control cell proliferation.

#### Disruptions in miRNA Processing

Recent studies have shown that impairments of the miRNA processing machinery are common in Wilms tumor and likely contribute to this disease. For example, a study by Torrezan et al. (2014) found mutations in miRNA processing genes in 33% of tumors, most commonly occurring in the *Drosha* gene, with other mutations in *DICER1, XPO5, DGCR8* and *TARBP2*. These results are supported by several other studies by Wu et al. (2013), Rakheja et al. (2014), Walz et al. (2015), Wegert et al. (2015), and Gadd et al. (2017). In Rakheja et al.’s study, they further examined the potential consequences of several of these mutations and found that Drosha mutations often led to a loss of RNAse IIIB activity, which prevented processing of pri-miRNAs, leading to a global reduction in mature miRNAs. *DICER1* mutations also frequently affected the RNAse IIIB domain; however, this mutation only affected processing of 5p miRNAs from precursors, as *DICER1* contains a second RNAse domain for 3p processing. As a result, this mutation led to a shift towards 3p miRNA maturation. These mutations have interesting consequences for global miRNA expression and most likely favor expression of oncogenic miRNAs or reduce expression of miRNAs with tumor-suppressive effects. In line with this, the let-7 family is predominantly 5p-derived, and lower expression of several of its 5p members was found in both *Drosha* and *DICER1* mutants in two of these studies (Rakheja et al., 2014; Walz et al., 2015). Additionally, the miR-200 family was found downregulated in Wilms tumors with mutated miRNA processing genes, which is known to regulate the mesenchymal-to-epithelial transition and has been associated with highly aggressive forms of cancer (Ceppi and Peter, 2014; Walz et al., 2015). The functional role of several oncomiRs has been investigated in detail within the context of pediatric solid tumors and is discussed in the following section.

#### The miR-17∼92 Cluster is a Downstream Effector of Oncogenic Transcription Factors

The miR-17∼92 cluster is expressed during normal development of the brain, heart, lungs, and immune system (Koralov et al., 2008; Ventura et al., 2008; Bian et al., 2013; Chen et al., 2013) and is known to regulate critical genes involved in cell growth, proliferation, and apoptosis. This cluster is comprised of six different miRNAs that are co-expressed, including miR-17, miR-18a, miR-19a, miR-19b-1, miR-20a, and miR-92-1. Dysregulation of the miR-17∼92 cluster has been shown in several pediatric solid tumors including neuroblastoma, Wilms tumor, retinoblastoma, and osteosarcoma, where a higher expression generally correlates with a poorer prognosis (Chen and Stallings, 2007; Baumhoer et al., 2012; Li et al., 2014). The miR-17∼92 cluster is particularly interesting due to its regulation by the transcription factor MYC and its homologue MYCN, where it seems to act as a mediator for some of MYC/MYCN’s oncogenic effects (Schulte et al., 2008). Other transcription factors known to target the miR-17∼92 cluster include members of the E2F family and STAT3 (Mogilyansky and Rigoutsos, 2013).

Several studies have demonstrated that the miR-17∼92 cluster regulates many downstream components of the transforming growth factor beta (TGF-β) pathway, which is known to participate in a variety of cellular process such as differentiation, proliferation, and immune cell activation. A study by Fontana et al. (2008) demonstrated that in neuroblastoma, miR-17 and miR-20a downregulate the cyclin-dependent kinase inhibitor p21, which is activated by TGF-β. p21 plays a key role in the inhibition of cell cycle progression by blocking the transition from G1 to S phase, and its deregulation leads to uncontrolled cell growth. Additionally, Fontana et al. (2008) showed that miR-17-5p regulated another downstream component of TGF-β, the pro-apoptotic factor Bcl-2 interacting mediator (BIM). Mestdagh et al. (2010) later investigated miR-17∼92 regulation of the TGF-β pathway in more depth and identified miR-17 and miR-20a as regulators of TGFBR2 and miR-18a as a regulator of SMAD2 and SMAD4, both signal transducers for TGF-β receptors. miR-18a and miR-19a have also been shown to repress estrogen receptor (ESR1) expression, and prolonged knockdown of miR-18a induced morphological differentiation of SK-N-BE neuroblastoma cells. Interestingly, the TGF-β pathway interacts with ESR1 signaling via several of the SMADs (Band and Laiho, 2011), suggesting a complex interplay between miR-17∼92 and its targeted pathways necessary for fine-tuning differentiation during neuronal development—a balance that is disrupted when miR-17∼92 is overexpressed. While no studies have investigated in detail the interaction between the miR-17∼92 cluster and TGF-β pathway in Wilms tumor, the TGF-β pathway has been implicated in Wilms tumor development. In contrast with neuroblastoma, the TGF-β pathway appears to function as a promoter of Wilms tumor progression, and TGF-β is highly expressed in primary tumors, even more so in metastatic tumors. This multifaceted behavior of the TGF-β pathway has been shown in other cancers and implies that the pathway’s influence is specific to the tumor it is activated in.

The E2F family of transcription factors serve an important role in cell cycle control as their expression can cause cells to enter the G1 phase to initiate cell division (Chen et al., 2009). Several members, including E2F1, E2F2, and E2F3, all regulate miR-17∼92 expression. In a study by Kort et al. (2008) a member of the E2F family of transcription factors, E2F3, was shown to be exclusively expressed in Wilms tumor and not in other types of kidney tumors. In line with this, they compared expression of the miR-17∼92 miRNAs in Wilms tumor samples to other renal tumor subtypes and found them all to be upregulated. They were also able to show a correlation between E2F3 expression and the stage of Wilms tumor, where it was highest in late-stage metastatic tissues. In retinoblastoma, an early study investigating the miR-17∼92 cluster identified that one of its members, miR-20a, participates in an autoregulatory feedback loop with E2F2 and E2F3 (Sylvestre et al., 2007), as they found both transcription factors are themselves downregulated by miR-20a. The authors suggested that this autoregulation was critical in preventing expression of excessive amounts of E2F transcription factors. Given that MYC/MYCN and E2F have previously been shown to induce each other’s expression, miR-20a appears to play an important role in keeping this positive feedback loop in check (Leone et al., 1997; Strieder and Lutz, 2003). Therefore, it is easy to see how disruption in one or more of these regulatory elements could lead to uncontrolled expression of these proliferative and anti-apoptotic signals.

A later study by Conkrite et al. (2011) investigated miR-17∼92 in retinoblastoma and revealed that this cluster was capable of driving retinoblastoma formation in *RB1/p107*-deficient mice. *RB1* plays a key role in inhibiting cell cycle progression, and germline mutations of this gene can lead to familial retinoblastoma formation (Friend et al., 1986; Classon and Harlow, 2002). *RB1*’s protein product, pRB, inhibits E2F transcription factors by binding and inactivating them, and its absence enables miR-17∼92–driven tumor formation.

The miR-17∼92 cluster also plays a role in driving tumor progression and metastasis in osteosarcoma (Li et al., 2014). A recent study by Yang et al. (2018) identified QKI2 as a regulatory target of the miR-17∼92 cluster. QKI proteins have previously been shown to inhibit β-catenin and induce differentiation in colon cancer. Yang at el. demonstrated that miR-17∼92 downregulated QKI2, causing upregulation of β-catenin, leading to increased proliferation, invasion, and migration in osteosarcoma (Yang et al., 2018). Additionally, miR-20a has previously been shown to downregulate Fas expression, which is a cell surface marker that interacts with FasL to induce apoptosis in the lungs, where osteosarcoma almost exclusively metastasizes to (Huang et al., 2012).

The miR-17∼92 cluster plays a tumorigenic role in a number of pediatric solid tumors including neuroblastoma, Wilms tumor, retinoblastoma, and osteosarcoma. The use of the miRNA pathway by transcription factors such as the MYC and E2F families enables them to target a wide range of genes and immediately effect gene expression at the post-transcriptional level. Continued research into how miRNAs may operate as oncogenic drivers will likely expand the repertoire of potential drug targets available to us.

#### Let-7 Dysregulation is a Feature in Many Pediatric Solid Tumors

The let-7 family of miRNAs are among the most well-characterized tumor suppressors due to their frequent downregulation in cancers. In total, there are 12 members of the let-7 family located across eight different chromosomes; however, in most cells, only a selection of these miRNAs will be expressed (Balzeau et al., 2017). let-7 miRNAs are important in regulating the cell cycle and maintaining cells’ differentiated state by targeting a wide range of genes with known roles in cancer biogenesis such as *MYC/MYCN, RAS, CDK6*, and *HMGA2* (Buechner et al., 2011; Wu et al., 2015).

let-7 is regulated by the LIN28 proteins, LIN28A and LIN28B, which mediate uridylation, prevent processing of the let-7 precursor, and are important for maintaining pluripotency in cells (Lehrbach et al., 2009; Balzeau et al., 2017). Both *Lin28* genes contain let-7 target sites and participate in a double-negative feedback loop with let-7 (Yin et al., 2017). Overexpression of *Lin28* tends to drive cells towards oncogenesis and is a common feature in cancers. In a study by Urbach et al. (2014), *Lin28b* overexpression was found in approximately 30% of Wilms tumors. Additionally, they found overexpression of *Lin28* could induce tumor formation in specific renal intermediates and that restoration of let-7 activity could reverse this effect in mice. Similar examples have been shown in mouse models, where *Lin28b* overexpression can drive hepatoblastoma and hepatocellular carcinoma in the liver and neuroblastoma in the neural crest (Molenaar et al., 2012; Nguyen et al., 2014). Molenaar et al. (2012) investigated Lin28b in neuroblastoma and demonstrated that Lin28b could enhance MYCN protein levels via let-7 regulation. However, a later study by Powers et al. (2016) showed that Lin28b expression was redundant in certain MYCN-amplified neuroblastoma cells, as overexpression of the MYCN transcript could function as a miRNA sponge for let-7, thereby negating their effect regardless of expression level. Powers et al. showed that most neuroblastomas were characterized by a loss of let-7 with either MYCN overexpression or chromosomal loss of arm 3p or 11q, where several let-7 miRNAs are located (**Figure 1A**). The authors noted that these events were generally mutually exclusive and suggested that the presence of one event alleviated selective pressure for the other.

A study by Di Fiore et al. (2016) revealed that let-7d could promote and suppress tumor formation within the same system. In this study, they found that let-7d overexpression in osteosarcoma cells reduced several stemness genes, including *Lin28b*, *HMGA2*, *Oct3/4*, and *SOX2*, and could elicit the mesenchymal-to-epithelial transition with upregulation of the epithelial marker E-cadherin and downregulation of mesenchymal markers N-cadherin and vimentin. However, they also found that let-7d enhanced cell migration and invasion, presumably by acting via the TGF-β pathway, which is known to promote this behavior. let-7d strongly increased versican VI expression, which has previously been shown to activate the TGF-β pathway in osteosarcoma (Li S. et al., 2014).

In Ewing sarcoma, Hameiri-Grossman et al. (2015) found that let-7 downregulated the *Ras* oncogene, as well as the transcription factor HIF-1a, to reduce EWS-FLI-1 expression (**Figure 1B**). EWS-FLI-1 is a hybrid transcript that results from a translocation event involving EWS and FLI1, and translocation events such as this are present in nearly all Ewing sarcoma cases and are believed to drive the disease (Delattre et al., 1994).

Loss of Let-7 plays a key role in many pediatric solid tumors as its loss enables expression of transcription factors and other genes that participate in oncogenesis. This has been emphasized in neuroblastoma, where it has been suggested that loss of let-7 function is an essential event in tumor development and positions the miRNA pathway as a central player in pediatric solid tumors.

#### miR-9 Has Been Shown to Play Oncogenic and Tumor-Suppressive Roles in Different Pediatric Tumors

miR-9 is a highly conserved miRNA involved in several different cellular processes including cell proliferation, differentiation, and migration. Early studies revealed miR-9 to be highly expressed in the brain and play a role both during development and in the adult brain; however, miR-9 has also been associated with many cancers outside the brain, acting as an oncogene or tumor suppressor (Coolen et al., 2013). Mir-9 is upregulated by MYC/MYCN and plays a role in promoting tumor growth and metastasis in several cancers including breast cancer, osteosarcoma, and rhabdomyosarcoma, where it is often overexpressed (Iorio et al., 2005; Luo et al., 2017) However, in other cancers such as neuroblastoma, miR-9’s role is less clear, and studies have argued for oncogenic and tumor suppressor functions (Laneve et al., 2007; Zhi et al., 2014).

The role of miR-9 in osteosarcoma appears to be in promoting cell growth and metastasis (Zhu et al., 2015; Qi et al., 2016). In a study by Zhu et al. (2015), miR-9 knockdown suppressed cell growth and migration of osteosarcoma cells. They were also able to show that miR-9 downregulated RB1 via the Grap2 and cyclin D interacting protein (GCIP), thereby promoting E2F-mediated cell division (**Figure 1C**). Similar behavior has been observed in the alveolar subtype of rhabdomyosarcoma, where miR-9 contributes to increased cell proliferation and migration (Missiaglia et al., 2017). In this study by Missiaglia et al. (2017), miR-9 was shown to be induced by the PAX3/FOXO1 fusion gene via MYCN, which is specific to this subtype of rhabdomyosarcoma.

In neuroblastoma, miR-9 expression has been shown to be both up- and downregulated in different studies. An early study by Laneve et al. (2007) showed that miR-9 was downregulated in 50% of primary neuroblastoma samples, and follow-up experiments demonstrated that miR-9 could act together with miR-125a and miR-125b to suppress cell proliferation by targeting a truncated isoform of the neurotrophin receptor tropomyosin-related kinase C (trkC) (**Figure 1D**). However, a later study by Ma et al. (2010) found miR-9 to be a target of MYCN and that miR-9 expression correlated with MYCN and metastatic status in neuroblastoma tumors. In this same study (albeit in breast cancer cells), Ma et al. also demonstrated that miR-9 suppressed E-cadherin to activate β-catenin and promote the epithelial-to-mesenchymal transition. Mir-9 is frequently involved in promoting cell migration; however, its absence has also been shown to produce different responses such as cell cycle arrest or apoptosis in neurons depending on their origin (Bonev et al., 2011). The contradictory behavior seen with studies of miR-9 highlight the diverse roles that individual miRNAs can play, and more comprehensive studies are needed to identify the relevant contextual influences on miRNA behavior.

#### miR-34 Is a Key Regulator of the Cell Cycle and Drug Resistance in Pediatric Solid Tumors

The miR-34 family has garnered significant interest since its members were discovered to be direct transcriptional targets of the tumor suppressor and transcription factor p53 (Hermeking, 2010). The miR-34 family consists of three miRNAs encoded by two genes, *mir-34a* and *mir-34b/c*. All three miRNAs play a key role in regulating apoptosis and the cell cycle by inducing G1 phase arrest. One of the more interesting facts about *miR-34a* and *miR-34b/c* is their genomic locations, which are located on chromosomes 1p36 and 11q23, respectively, regions that are frequently lost in pediatric solid tumors (Ruteshouser et al., 2005; Wittmann et al., 2007). In particular, loss of 1p36 occurs in 20–30% of neuroblastoma cases and correlates with MYCN amplification (Caron et al., 1993; Maris et al., 1995), whereas loss of 11q23 in occurs in approximately 40% of cases but almost never occurs with MYCN amplification (**Figure 1A**) (Guo et al., 1999; Attiyeh et al., 2005). miR-34 members are also regulators of the MYC family, as miR-34a is known to regulate MYCN and miR-34b and mir-34c to regulate c-MYC (Wei et al., 2008).

Studies on mir-34a expression have identified frequent downregulation in neuroblastoma, osteosarcoma, and hepatoblastoma (Jiao et al., 2016). miR-34a is itself considered a tumor suppressor due to its involvement in cell cycle arrest and apoptosis (De Antonellis et al., 2014). In neuroblastoma, Cole et al. (2008) investigated the growth-inhibitory effects of several miRNAs mapping to common chromosomal aberrations by overexpressing them in cell lines. In most cases, overexpression did not lead to a noticeable change in phenotype; however, miR-34a and miR-34c induced significant growth inhibition in cell lines with 1p36 deletion. Growth inhibition and suppression of metastasis by miR-34a have also been shown in osteosarcoma by several studies, where members of key proliferative signal transduction pathways such as c-Met, DUSP1, and Eag1 were identified as regulatory targets (Yan et al., 2012; Wu X. et al., 2013; Gang et al., 2017). The miR-34 family also targets several members of the Notch signaling pathway, which has been linked to both oncogenic and tumor-suppressive roles depending on the cellular context. In osteosarcoma, activation of the Notch pathway is known to contribute to tumor growth, and miR-34a–mediated downregulation of this pathway likely contributes to its tumor-suppressive role. However, in Ewing sarcoma, a recent study investigating miR-34b suggested that it could act as an oncogene, promoting proliferation, migration, and invasion through Notch1 repression (Lu Q. et al., 2018). Prior studies have shown correlations between high mir-34a expression and patient survival, which would indicate a tumor-suppressive role for mir-34a (Nakatani et al., 2012; Marino et al., 2014). It is unclear why miR-34a and miR-34b would display contrasting effects given their shared targets, and further investigation is needed.

Several studies by Pu et al. (2016)and Pu et al. (2017) have suggested that miR-34a may also play a role in promoting multidrug resistance in osteosarcoma. In these studies, they found that miR-34a-5p enhanced multidrug resistance through downregulation of the *CD117* and *AGTR1* genes *in vitro*. CD117 is often highly expressed in drug-resistant tumors and is commonly used as a marker for stemness (Adhikari et al., 2010). In contrast, Nakatani et al. found that miR-34a increased chemosensitivity in Ewing sarcoma (Nakatani et al., 2012).

#### Other miRNAs Involved in Multiple Pediatric Solid Tumors

A substantial number of other miRNAs have been discovered with functional implications in multiple pediatric solid tumors. One such miRNA is miR-125b, which typically exhibits tumor-suppressive properties in cancers such as neuroblastoma, osteosarcoma, and Ewing sarcoma, where it is commonly dysregulated (Laneve et al., 2007; Li J. et al., 2014; Xiao et al., 2019). Previously, it was mentioned that miR-125b participates in a network with miR-125a and miR-9, regulating expression of a truncated trkC isoform to control neuroblastoma growth and differentiation (Laneve et al., 2007; Le et al., 2009). In osteosarcoma, miR-125b was found to regulate STAT3 by downregulating MAP kinase kinase 7 (MKK7), which inactivates STAT3 via dephosphorylation (Xiao et al., 2019). Loss of miR-125b and consequent overexpression of MKK7 led to increased tumor formation and poorer prognosis. In Ewing sarcoma, miR-125b is involved in regulating the PI3K signaling pathway; could inhibit cell proliferation, migration, and invasion; and induce apoptosis through suppression of PIK3CD (Li J et al., 2014). Conversely, in retinoblastoma, miR-125b is overexpressed and has shown oncogenic properties by promoting cell proliferation and migration and inhibiting apoptosis (Bai et al., 2016). Conflicting behavior with miR-125b has been observed in many other cancers, which suggests that its role is highly dependent on cell identity (Sun et al., 2013).

miR-124 has been widely reported to act as a tumor suppressor by inhibiting cell growth and metastasis and acts as a key mediator of differentiation in several pediatric solid tumors (Peng et al., 2014; Feng et al., 2015; Zhao et al., 2017). In neuroblastoma, miR-124a increased the proportion of differentiated cells possessing neurite outgrowths (Le et al., 2009). In retinoblastoma, miR-124 participates in a regulatory network with lncRNAs Malat1 and XIST, which both function as oncogenes by enhancing growth and metastasis through downregulation of miR-124 (Liu S. et al., 2017; Hu et al., 2018). miR-124 itself was shown to target STAT3 to inhibit cell proliferation, migration, and invasion (Liu S. et al., 2016). In Ewing sarcoma, miR-124 expression is suppressed, and expression was found to reduce growth and metastasis *via* downregulation of mesenchymal genes such as *SLUG* and cyclin D2 (*CCND2*) (Li et al., 2017). Finally, in osteosarcoma, retinoblastoma, and Ewing sarcoma, miR-143 has been found to be dysregulated (De Vito et al., 2012; Li S. et al., 2014; Wang et al., 2016; Sun et al., 2018). For example, Li et al. investigated miR-143 function in osteosarcoma and showed that miR-143 participated in the TGF-β pathway by targeting versican, and TGF-β could reduce miR-143 expression to promote cell migration and invasion (Li S. et al., 2014). FOS-like antigen 2 (FOSL2) was also identified as a miR-143 target, which enhanced cell proliferation, migration, and invasion in the absence of miR-143 (Sun et al., 2018). Additional miRNA studies have been listed in **Table 1**.

**Table 1:** miRNAs that have been shown to exhibit oncogenic or tumor-suppressive effects through functional studies in various pediatric solid tumors.

Cancer	Oncogenic miRNAs/clusters	Comments
Neuroblastoma	mir-15 (Xin et al., 2013), mir-17~92 (Fontana et al., 2008; Loven et al., 2010; Mestdagh et al., 2010), miR-93 (Chakrabarti et al., 2012), miR-380 (Swarbrick et al., 2010), miR-558 (Shohet et al., 2011; Qu et al., 2015)	mir-17~92 dysregulation is common in MYCN-amplified neuroblastomas.
Osteosarcoma	let-7d (Di Fiore et al., 2016), miR-9 (Zhu et al., 2015; Qi et al., 2016), miR-17~92 (Huang et al., 2012; Li X. et al., 2014; Lu et al., 2018a; Yang et al., 2018b), miR-34a (Pu et al., 2016; Pu et al., 2017), miR-214 (Rehei et al., 2018)	
Retinoblastoma	miR-17~92 (Conkrite et al., 2011; Nittner et al., 2012; Jo et al., 2014), miR-125b (Bai et al., 2016)	Loss of RB1 function may enable mir-17~92–mediated oncogenicity.
Wilms tumor	miR-19b (Liu G.-L. et al., 2017), miR-483 (Veronese et al., 2010; Liu M. et al., 2013), miR-1180 (Jiang and Li, 2018)	
Hepatoblastoma	miR-492 (von Frowein et al., 2018)	
Ewing sarcoma	mir-17~92 (Schwentner et al., 2017), miR-20b (Kawano et al., 2017), miR-34b, b (Lu et al., 2018b) miR-130b (Satterfield et al., 2017)	EWS-FLI-1 may upregulate mir-17~92 and miR-34b.
Cancer	Tumor-suppressive miRNAs/clusters	Comments
Neuroblastoma	let-7 (Buechner et al., 2011; Molenaar et al., 2012; Hennchen et al., 2015; Powers et al., 2016), mir-7-1 (Chakrabarti et al., 2012), mir-9 (Laneve et al., 2007), mir-10 (Foley et al., 2011), miR-34a (Welch et al., 2007; Cole et al., 2008; Tivnan et al., 2011), miR-34c (Cole et al., 2008), miR-101 (Buechner et al., 2011), miR-124a (Le et al., 2009), miR-125 (Laneve et al., 2007; Le et al., 2009), miR-145 (Zhang et al., 2014), miR-184 (Chen and Stallings, 2007; Foley et al., 2010; Tivnan et al., 2010), miR-193b (Roth et al., 2018), miR-202 (Buechner et al., 2011), miR-203 (Zhao et al., 2015), miR-449 (Buechner et al., 2011), miR-542 (Bray et al., 2011), miR-584 (Xiang et al., 2015), miR-591 (Shohet et al., 2011)	let-7 and mir-34 are regulators of the MYCN oncogene.
Osteosarcoma	let-7d (Di Fiore et al., 2016), miR-1 (Novello et al., 2013), miR-34 (Yan et al., 2012; Wu et al., 2013b; Gang et al., 2017; Wen et al., 2017), miR-125b (Le et al., 2009; Xiao et al., 2019), miR-133b (Novello et al., 2013), miR-134 (Thayanithy et al., 2012), miR-138 (Zhu et al., 2016), miR-143 (Li et al., 2014c; Sun et al., 2018), miR-195 (Han et al., 2015), miR-223 (Dong et al., 2016), miR-363 (Wang K. et al., 2018), miR-369 (Thayanithy et al., 2012), miR-382 (Thayanithy et al., 2012), miR-451 (Yuan et al., 2015), miR-454 (Niu et al., 2015), miR-485 (Du et al., 2018), miR-544 (Thayanithy et al., 2012), miR-590 (Wang W.T. et al., 2018), miR-708 (Chen and Zhou, 2018), miR-2682 (Zhang et al., 2018b)	miR-34 suppresses tumor growth in osteosarcoma but may also contribute to drug resistance.
Retinoblastoma	miR-101 (Lei et al., 2014), miR-124 (Liu S. et al., 2016), miR-143 (Wang et al., 2016)	STAT3 is a target of miR-124.
Rhabdomyosarcoma	miR-1 (Rao et al., 2010; Li et al., 2012), mir-22 (Bersani et al., 2016), miR-29 (Li et al., 2012), miR-133a (Rao et al., 2010), miR-206 (Li et al., 2012; Missiaglia et al., 2010)	miR-1 appears to play a key role in differentiation of several sarcomas.
Wilms tumor	let-7 (Urbach et al., 2014), miR-16 (Chen W. et al., 2018), miR-34a (Chen W. et al., 2018), mir-92a (Zhu et al., 2018b), miR-613 (Wang et al., 2017a)	miR-92a was shown to act as a tumor suppressor unlike what is observed in other pediatric solid tumors.
Hepatoblastoma	miR-26 (Zhang et al., 2018d)	miR-26 was shown to repress LIN28B in hepatoblastoma.
Ewing sarcoma	let-7 (Hameiri-Grossman et al., 2015; Kawano et al., 2015), miR-16 (Kawano et al., 2015), miR-22 (Parrish et al., 2015), miR-29b (Kawano et al., 2015), miR-30a (Franzetti et al., 2013), miR-30d (Ye et al., 2018), miR-31 (Karnuth et al., 2014), miR-34a (Nakatani et al., 2012; Ventura et al., 2016), miR-124 (Li et al., 2017), miR-125b (Li et al., 2014b), miR-143 (De Vito et al., 2012), miR-145 (Riggi et al., 2010; De Vito et al., 2012), miR-185 (Zhang et al., 2018c), miR-193b (Moore et al., 2017), miR-708 (Robin et al., 2012)	Several miRNAs such as let-7 and miR-145 are downregulated by EWS-FLI-1.

#### miRNAs Regulate All Aspects of Tumorigenesis

Widespread dysregulation of miRNAs is observed in many pediatric solid tumors, and functional studies have demonstrated that many of these miRNAs can drive or repress oncogenic pathways responsible for cell proliferation, apoptosis, angiogenesis, metastasis, and drug resistance. Importantly, miRNAs such as let-7 and miR-34 play a vital role in pediatric solid tumors by regulating established oncogenic transcription factors such as the MYC and E2F families (Wei et al., 2008; Buechner et al., 2011). Other miRNAs, such as the miR-17∼92 cluster and miR-9, serve as downstream effectors for these transcription factors, although their exact role in tumorigenesis seems to depend on the overall transcriptional landscape (Schulte et al., 2008; Ma et al., 2010). In some cases, viewing miRNAs as oncogenes or tumor suppressors likely represents an oversimplification of their role in cancer, and a better understanding of their participation in oncogenic networks will be needed to clarify their exact contributions.

## Long Non-Coding RNAs Regulate Oncogenic Pathways in Pediatric Solid Tumors

For a long time, it was believed that the human genome was mostly comprised of “junk” DNA, despite pervasive transcription of much of the genome outside of protein-coding genes and other known RNAs at the time (Prensner and Chinnaiyan, 2011). Originally thought of as transcriptional noise, lncRNAs have now emerged as functional regulators of nearly all essential cellular processes including growth, differentiation, cell state maintenance, apoptosis, splicing, and epigenetic regulation. The first lncRNA, H19, was discovered in 1990 where an RNA molecule was found spliced and polyadenylated in a manner typical of mRNAs; however, it lacked an open reading frame and was believed to function as an untranslated RNA molecule (Brannan et al., 1990).

Often, lncRNAs participate within protein complexes and can operate as scaffolds, guides, decoys, or allosteric regulators. Many lncRNAs function as epigenetic regulators by interacting with proteins involved in chromatin remodeling and DNA methylation. Frequently, these lncRNAs will be cis-acting and regulate the regions near their transcribed location; however, some are trans-acting. Other lncRNAs function as competing RNAs (ceRNAs), which contain miRNA binding sites in a similar manner to mRNAs in order to compete and reduce the activity of miRNAs.

Several studies have investigated lncRNA expression in pediatric tumors and have successfully identified unique expression profiles in different cancers and tumor subtypes (Mitra et al., 2012; Brunner et al., 2012; Dong et al., 2014; Sahu et al., 2018). For example, Dong et al. (2014) compared hepatoblastoma samples to normal liver tissue in patients and found 2,736 differentially expressed lncRNAs. A study by Pandey et al. (2014) found 24 lncRNAs that could distinguish low- and high-risk neuroblastoma tumors. In a more recent study, Sahu et al. (2018) identified 16 differentially expressed lncRNAs that could be used to predict event-free survival with greater accuracy than other commonly used clinical risk factors. Mechanistic studies into many of these lncRNAs have revealed that they frequently act as an additional layer of regulation within established oncogenic networks involving protein-coding genes and miRNAs. While the field of lncRNAs is still relatively young, many studies have emerged that suggest that lncRNAs are far more integrated into existing gene networks than what has previously been appreciated (**Figure 1**). In the following section, the roles of some of the better-characterized lncRNAs in pediatric solid tumors will be discussed.

### Malat1 Is Induced by MYCN in Neuroblastoma and Competes With Many miRNAs

One of the earliest lncRNAs to be associated with disease was Malat1 (metastasis-associated lung adenocarcinoma transcript 1), which was shown to associate with metastatic tumors in non–small cell lung cancer patients (Ji et al., 2003). Malat1 is abundantly expressed and highly conserved across species, unlike many other lncRNAs, and displays remarkably diverse functions in cellular processes including alternative splicing, nuclear organization, and epigenetic modulation. Studies have suggested an important role for Malat1 in brain development, as it is highly expressed in neurons and its depletion has been shown to affect synapse and dendrite development (Bernard et al., 2010; Chen et al., 2016). However, its importance has been questioned as other studies have found that Malat1-KO mice are viable with no discernable change in phenotype (Nakagawa et al., 2012; Zhang et al., 2012).

In addition to lung cancer, Malat1 is known to contribute to metastasis in other common types of cancer including hepatocellular carcinoma and bladder cancer, with evidence that it acts through induction of the epithelial-to-mesenchymal transition (Ying et al., 2012; Li G. et al., 2014; Yang et al., 2017). The role of Malat1 in several pediatric cancers has also been explored in recent studies. In neuroblastoma, Tee et al. (2014) recently identified a regulatory network involving N-Myc, Malat1, and the histone demethylase JMJD1A. They found that N-Myc upregulated JMJD1A via direct binding of its promoter region and that JMJD1A could demethylate histone H3K9 near the promoter region of Malat1, leading to its upregulation. MYCN-mediated upregulation of Malat1 provides one mechanism in which its amplification can lead to increased metastasis in neuroblastoma patients. Another study by Bi et al. (2017) also demonstrated that Malat1 regulated Axl expression, a transmembrane receptor tyrosine kinase, which is known to activate pathways involved in cell proliferation, survival, and migration. In osteosarcoma, Dong et al. (2015) demonstrated that Malat1 was highly expressed and could activate the PI3K/Akt pathway to promote proliferation and invasion.

Malat1 is known to interact with many miRNAs implicated in cancer. In osteosarcoma, several studies have shown Malat1 can function as a ceRNA for different miRNAs (Wang et al., 2017b; Liu K. et al., 2017b; Sun and Qin, 2018). miR-140-5p is a tumor suppressor that downregulates HDAC4, a histone deacetylase that contributes to tumorigenesis, and competitive binding by Malat1 with miR-140-5p was shown to increase HDAC4 activity (Sun and Qin, 2018). Malat1 was also shown to compete with miR-144-3p binding to ROCK1/ROCK2, promoting proliferation and metastasis (Wang et al., 2017b). In a similar manner, Liu K. et al. (2017) found that Malat1 could regulate cell growth through high-mobility group protein B1 (HMGB1) via ceRNA activity with miR-142-3p and miR-129-5p. Finally, in retinoblastoma, Malat1 downregulated miR-124 activity, leading to activation of the transcription factor SLUG, which is also targeted by miR-124 (Liu S. et al., 2017). SLUG has a known role in the epithelial-to-mesenchymal transition by suppressing E-cadherin via the Wnt/B-catenin pathway (Prasad et al., 2009).

In addition to interactions with miRNAs, Malat1 has also been shown to be processed directly by the Drosha–DGCR8 microprocessor complex through binding sites in the 5’ end of the transcript (MacIas et al., 2012). lncRNAs such as Malat1 cooperate with the miRNA pathway and a number of transcription factors and epigenetic factors to form a complex network responsible for regulating tumorigenesis. The capacity for Malat1 to drive proliferation and metastasis in pediatric solid tumors suggests that dysregulation of any of these regulatory components can be sufficient for the development of cancer and highlights the value of further research into the relatively new field of lncRNAs.

### H19: lncRNA Dysregulation via Loss of Imprinting may Contribute to Tumorigenesis

H19 is a paternally imprinted gene that is typically expressed exclusively from the maternal allele. Early reports suggested that H19 functioned as a tumor suppressor capable of inhibiting cell growth (Hao et al., 1993; Zhang et al., 1993; Casola et al., 1997; Fukuzawa et al., 1999). Studies in childhood solid tumors such as hepatoblastoma, Wilms tumor, and embryonic rhabdomyosarcoma supported this idea, as all three cancers often exhibited reduced H19 expression and had frequently lost the maternal 11p15 chromosomal region housing this gene (Fukuzawa et al., 1999). Other studies, in osteosarcoma and retinoblastoma, suggested an oncogenic role for H19, as its upregulation and loss of imprinting were commonly seen (Chan et al., 2014; Li L. et al., 2018). This observation was also seen in many other cancers including breast cancer (Lottin et al., 2002). Recently, the Hedgehog signaling pathway, a regulator of differentiation known to participate in cancer development and metastasis, was shown to induce H19 expression (Chan et al., 2014).

Understanding the exact function of H19 has proved difficult; however, it was known to sit downstream of the insulin growth factor 2 (IGF2) gene, a growth factor known to play a role in tumorigenesis. Early reports suggested interactions between IGF2 and H19, as loss of imprinting of either gene caused biallelic expression of the other gene (Ulaner et al., 2003). Ulaner et al. (2003) proposed a model for H19 and IGF2 involving a CCTF-binding site seated between the two genes, which could facilitate the blocking of IGF2 or transcription of H19 depending on its methylation status. However, this model suggested that H19 may simply serve as a marker for epigenetic disruptions and left open the question of what H19’s actual function is.

More recent studies have demonstrated a role for H19 in epigenetic regulation. H19 binds to several epigenetic regulators including S-adenosylhomocysteine hydrolase (SAHH), methyl-CpG–binding domain protein 1 (MBD1), and enhancer of zeste homolog 2 (EZH2) (Raveh et al., 2015; Zhou et al., 2015). H19 was found to inhibit SAHH, which led to downregulation of DNMT3B-mediated methylation. MBD1 binds methylated DNA and recruits other proteins to mediate transcriptional repression or histone methylation, and H19 was shown to recruit this protein to several genes including IGF2 (Monnier et al., 2013). Finally, EZH2 is a histone methyltransferase that forms part of the Polycomb repressive complex 2 (PRC2) (Sauliere et al., 2006; Zhou et al., 2015).

H19 also plays a role in maintaining cells in an undifferentiated state by associating with the KH-type splicing regulatory protein (KSRP). When multipotent mesenchymal cells were induced, H19 was found to dissociate with KSRP to promote several of its functions including the decay of unstable mRNAs and increasing the expression of specific miRNAs involved in proliferation and differentiation though association with Drosha and Dicer.

H19’s role in cancer has been emphasized by studies highlighting its relationship to the tumor suppressor p53. The H19 locus reciprocally regulates p53, as p53 suppresses H19 transcription and H19 can inactivate p53 by directly interacting with it (Yang et al., 2012). Notably, H19 also encodes for a miRNA in its first exon, miR-675, which suppresses p53 and several other targets including Rb, Igf1r, and several SMAD and cadherin genes. In the absence of functional p53, H19 was shown to promote tumor proliferation and survival under hypoxic conditions. Later studies in colorectal cancer showed that H19 could induce EMT by acting as a ceRNA (Liang et al., 2015). ceRNA function has recently been shown in a retinoblastoma study, targeting the mir-17∼92 cluster (Zhang A. et al., 2018a). In this study, they found that H19 contained seven functional binding sites for mir-17∼92 and was able to sponge mir-17∼92 activity. This led to a de-repression of genes such as *p21* and STAT3 targets *BCL2, BCL2L1*, and *BIRC5*.

In a review by Raveh et al. (2015) it was proposed that H19 may behave differently in a manner that was dependent on the developmental stage of the cell, which could explain the evidence suggesting both oncogenic and tumor-suppressive roles. Here, the authors found that H19 functioned as a promoter of differentiation during the embryonic period and that absence of H19 at this stage could leave cells vulnerable to forming cancer, thereby seemingly acting as a tumor suppressor. However, in adult cells, where it is not normally expressed, H19 could function as an oncogene by promoting tumor survival and metastasis (Matouk et al., 2015).

### TUG1 Regulates Transcription Factors Through Competition With miRNAs in Osteosarcoma

Recent studies have investigated the role of lncRNA TUG1 as a prognostic factor and ceRNA in osteosarcoma. Ma et al. (2016)identified a correlation between upregulation of TUG1 and poor prognosis and metastasis, which was also evident in plasma, and suggested a potential use as a biomarker for patients with osteosarcoma. TUG1 is known to act through ceRNA activity against a number of miRNAs including miR-9, miR-132, miR-144, miR-153, miR-212, and miR-335 (Xie et al., 2016; Cao et al., 2017; Wang et al., 2017a; Li G. et al., 2018; Li H. et al., 2018). These miRNAs are known to regulate pathways involved in proliferation, cell cycle control, migration, and apoptosis. For example, TUG1 was shown to mediate de-repression of the transcription factor POU class 2 homeobox1 (POU2F1) via downregulation of mir-9 (**Figure 1E**) (Xie et al., 2016). POU2F1 itself participates in various cellular processes including growth, metabolism, stem cell identity, and metastasis (Vázquez-Arreguín and Tantin, 2016). In another example by Cao et al. (2017) they found that TUG1 also regulates migration and the epithelial-to-mesenchymal transition via ceRNA action on miRNA-144-3p. miR-144-3p is a regulator of EZH2, and upregulation of EZH2 induced cell migration through the Wnt/β-catenin pathway (Cao et al., 2017). Studies have also demonstrated direct interactions between TUG1 and the Polycomb repressor complex; however, to our knowledge, this has not been investigated in pediatric solid tumors (Yang et al., 2011).

### Other Long Non-Coding RNAs in Pediatric Solid Tumors

In addition to those mentioned above, there are a number of other lncRNAs that have been identified as potential oncogenes or tumor suppressors involved in the pathogenesis of pediatric solid tumors (see **Table 2**) (Chen et al., 2017; Pandey et al., 2015).

**Table 2:** lncRNAs that play a role in pediatric solid tumors. OS, osteosarcoma; RB, retinoblastoma; NB, neuroblastoma; WT, Wilms tumor; HB, hepatoblastoma; RMS, rhabdomyosarcoma; ES, Ewing sarcoma.

Long Non-coding RNA	Cancer	Cellular Functions	References
Malat1	OS, RB, NB	Upregulates—proliferation, survival, migration, invasion.	(Tee et al., 2014; Dong et al., 2015; Bi et al., 2017; Liu K. et al., 2017; Liu S. et al., 2017; Wang K. et al., 2017; Sun and Qin, 2018)
H19	OS, RB, WT, HB, RMS	Upregulates—proliferation, survival. Regulates cell fate/differentiation.	(Zhang et al., 1993; Casola et al., 1997; Fukuzawa et al., 1999; Chan et al., 2014; Matouk et al., 2015; Raveh et al., 2015; Li L. et al., 2018)
TUG1	OS	Upregulates—proliferation, survival, migration.	(Ma et al., 2016; Xie et al., 2016; Cao et al., 2017; Wang S. et al., 2017; Li G. et al., 2018; Li H. et al., 2018)
HOTAIR	OS, RB	Upregulates—proliferation, survival, migration, invasion.	(Yang G. et al., 2018; Wang et al., 2019)
HOTTIP	OS	Upregulates—proliferation, resistance.	(Li et al., 2016)
SNHG12	OS	Upregulates—proliferation, migration.	(Ruan et al., 2016)
SNHG16	OS	Upregulates—proliferation, survival, migration, invasion.	(Su et al., 2019; Zhu et al., 2018a)
THOR	OS, RB	Upregulates—proliferation, migration.	(Chen K.S. et al., 2018; Shang, 2018)
PACER	OS	Upregulates—proliferation, invasion.	(Qian et al., 2016)
MFI2	OS	Upregulates—proliferation, survival, migration, invasion.	(Yin et al., 2016)
loc285194	OS	Downregulates—proliferation.	(Pasic et al., 2010)
TUSC7	OS	Downregulates—proliferation.	(Cong et al., 2016)
MEG3	OS, RB	Downregulates—proliferation, survival, invasion.	(Gao et al., 2017; Shi et al., 2018)
EWSAT1	OS, ES	Upregulates—proliferation, metastasis.	(Howarth et al., 2014; Sun et al., 2016)
XIST	RB	Upregulates—proliferation, survival.	(Hu et al., 2018)
DANCR	RB	Upregulates—proliferation, migration, invasion.	(Wang J. X. et al., 2018)
HOXA11-AS	RB	Upregulates—proliferation, survival.	(Han et al., 2019)
PANDAR	RB	Upregulates—survival.	(Sheng et al., 2018)
lncUSMYcN	NB	Upregulates—proliferation.	(Liu et al., 2014; Liu S. et al., 2016)
NBAT-1	NB	Downregulates—proliferation, invasion. Regulates cell fate/differentiation.	(Pandey et al., 2014)
CASC15-S	NB	Downregulates—proliferation, migration.	(Russell et al., 2015)
LINC00473	WT	Upregulates—proliferation, survival.	(Zhu et al., 2018b)
CRNDE	HB	Upregulates—proliferation, angiogenesis.	(Dong et al., 2017)
LINC01314	HB	Downregulates—proliferation, migration.	(Lv et al., 2018)

For example, in osteosarcoma, lncRNAs HOTAIR, SNHG16, SNHG12, THOR, PACER, MFI2, and HOTTIP have all been shown to promote tumor or cell growth (Li et al., 2016; Qian et al., 2016; Ruan et al., 2016; Yin et al., 2016; Chen W. et al., 2018; Su et al., 2019; Wang et al., 2019). HOTAIR is known to play a role in chromatin regulation by acting as a scaffold for PRC2 and lysine-specific histone demethylase 1 (LSD1), and can also act as a ceRNA for miR-217 (Gupta et al., 2010; Tsai et al., 2010; Wang et al., 2019). SNHG16 has been shown to act as a ceRNA for several miRNAs including miR-205 and miR-340 (Zhu C. et al., 2018; Su et al., 2019). Additionally, several lncRNAs are downregulated in osteosarcoma with potential tumor-suppressive activity such as loc285194, MEG3, and TUSC7 (Pasic et al., 2010; Cong et al., 2016; Shi et al., 2018). loc285194 has been identified as a transcriptional target of p53 and can downregulate miR-211 (Liu Q. et al., 2013). In another study, increased p53 expression and a decrease in cell proliferation and invasion were observed when MEG3 was overexpressed (Shi et al., 2018). Furthermore, MEG3 was found to be downregulated by another lncRNA, EWSAT1, which had previously been shown to enhance cell proliferation and metastasis in both osteosarcoma and Ewing sarcoma (Howarth et al., 2014; Sun et al., 2016).

In retinoblastoma, HOTAIR, THOR, and MEG3 appear to have a similar influence as seen in osteosarcoma, where they also acted as oncogenes (HOTAIR and THOR) or tumor suppressors (MEG3) (Gao et al., 2017; Shang, 2018; Yang G. et al., 2018). In the study examining HOTAIR in retinoblastoma, HOTAIR was shown to be engaged in a reciprocal regulatory loop with miR-613 and promoted cell proliferation and activation of the EMT, potentially through upregulation of N-cadherin, vimentin, and α‐SMA (Yang G. et al., 2018). Several lncRNAs have also been found acting as oncogenic ceRNAs including XIST, DANCR, and HOXA11-AS (Hu et al., 2018; Wang J. X. et al., 2018; Han et al., 2019). Finally, PANDAR is upregulated in retinoblastoma and may regulate cell proliferation and apoptosis via the Bcl-2/caspase-3 pathway (Sheng et al., 2018).

A number of studies have also suggested an important role for lncRNAs in neuroblastoma. For example, lncUSMYcN is an lncRNA that is frequently co-amplified alongside MYCN (Liu P. Y. et al., 2016). Liu et al. found that in neuroblastoma, lncUSMycN could upregulate MYCN through transcriptional activation of NCYM (a.k.a. MYCNOS), which codes for a protein that stabilizes MYCN (Suenaga et al., 2014). NCYM RNA has also been suggested to bind to the RNA-binding protein NonO, which is also known to upregulate MYCN expression (Liu et al., 2014; Liu P. Y. et al., 2016). Neuroblastoma associated transcript-1 (NBAT-1) is an epigenetic regulator that interacts with EZH2, and functions as a tumor suppressor due to its important role in neuronal differentiation (Pandey et al., 2014). Loss of NBAT-1 expression was found to increase cell proliferation and invasion (Pandey et al., 2014). Finally, an isoform of lncRNA CASC15, CASC15-S, was also implicated as a key element in neuronal differentiation, and low expression was associated with a poor outcome in patients (Russell et al., 2015).

In Wilms tumor, a study by Zhu et al. identified LINC00473 as an oncogenic lncRNA that is upregulated in unfavorable tumors (Zhu et al., 2018b). LINC00473 was shown to promote tumor growth and metastasis by acting as a ceRNA for the tumor suppressor miR-195 (Zhu et al., 2018b).

A study by Dong et al. identified 1757 upregulated and 979 downregulated lncRNAs comparing hepatoblastoma and normal tissues, suggesting that lncRNAs play a key role in this disease as well (Dong et al., 2014). The lncRNAs Colorectal Neoplasia Differentially Expressed (CRNDE) and LINC01314 have been investigated in more detail in hepatoblastoma (Dong et al., 2017; Lv et al., 2018). CRNDE is known to be frequently upregulated in hepatoblastoma, and knockdown of CRNDE activated the mTOR pathway and inhibited tumor growth and angiogenesis with a corresponding decrease in VEGFA and Ang-2 levels (Dong et al., 2017). LINC01314 was identified as a tumor suppressor, reducing proliferation and migration via downregulation of cell cycle proteins MCM7 and cyclin D1 (Lv et al., 2018).

## Concluding Remarks

It is now clear that both miRNAs and lncRNAs form integral parts of the biological networks known to be impaired in pediatric solid tumors. miRNAs such as let-7 and mir-34 are key regulators of many pediatric oncogenes including *MYC, MYCN, RAS*, and *MET* (Johnson et al., 2005; Wei et al., 2008; Buechner et al., 2011; Yan et al., 2012). Additionally, ncRNAs such as the miR-17∼92 cluster, mir-9, and Malat1 also serve as downstream effectors of MYC and MYCN (Schulte et al., 2008; Ma et al., 2010; Tee et al., 2014). Many more ncRNAs participate in these and other pathways to form a highly complex regulatory network essential for maintaining an optimal cell state (See **Tables 1** and **2**). ncRNA dysregulation offers an alternative mechanism to genetic mutations and DNA methylation whereby cell development and differentiation can be disturbed. Despite the relatively rare occurrence of mutations in pediatric solid tumors, copy number variations are common and often occur at regions of the genome that harbor ncRNAs with tumor-suppressive roles (Wei et al., 2008; Powers et al., 2016). Gene expression is often imprecise; however, miRNAs provide a layer of robustness, which helps ensure that biological networks respond appropriately to signals and remain functional despite an ever-increasing cellular disorder (Ebert and Sharp, 2012). lncRNAs, too, play a vital role in maintaining order by forming RNA–protein complexes and serving as ceRNA antagonists against miRNA-mediated repression, although much more work is needed in this field to fully comprehend their range of biological roles. Functional studies have revealed that dysregulation of ncRNAs is capable of driving progenitor cells towards oncogenesis. For example, this has been shown in retinoblastoma, where overexpression of the mir-17∼92 cluster could drive tumor formation in *RB/p107*-deficient mice (Conkrite et al., 2011).

While genome-wide association studies have revealed that miRNA processing is frequently disrupted in Wilms tumor, this has not been shown to the same extent in other pediatric solid tumors. However, genetic mutations of protein-coding genes are only one way in which disruptions of miRNA processing can be revealed. Most miRNA studies ignore the fact that a high proportion of expressed miRNAs are isoforms (isomiRs). isomiRs originating from the same miRNA gene can possess a great deal of functional variability, with differences in target acquisition or turnover rate that can have a significant impact on overall gene regulation. Studies focusing on isomiR expression will provide an additional layer of resolution to our understanding of miRNA dysregulation.

Recent developments in single-cell technology have revealed heterogeneity in gene expression profiles among individual cells in many cancers such as glioblastoma and neuroblastoma (Patel et al., 2014; Boeva et al., 2017). Such studies suggest that many tumors comprise different cellular subtypes with unique phenotypes such as growth rate, drug resistance, and metastatic potential, which demand a new way of approaching cancer treatments. miRNA expression in pediatric solid tumors may also be heterogenous; however, limitations in single-cell technologies have left this avenue relatively unexplored, and further developments are needed.

So far, ncRNA research has played a key role in advancing our understanding of the mechanisms behind pediatric solid tumor development. Evidence supports an active role for ncRNAs in cancer that extends beyond mere passengers. However, continued research is needed to fully comprehend the molecular events leading to the development of cancer and unlock new possibilities for drug targets and biomarkers, which will ultimately lead to a better outcome for patients afflicted by these diseases.

## Author Contributions

CS collected the information and wrote the review. DC and GH provided guidelines, consulted, and edited the manuscript.

## Funding

This work was supported by the Australian Research Council DP180100120 project grant.

## Conflict of Interest Statement

The authors declare that the research was conducted in the absence of any commercial or financial relationships that could be construed as a potential conflict of interest.
